# Konjac glucomannan-templated synthesis of three-dimensional NiO nanostructures assembled from porous NiO nanoplates for gas sensors†

**DOI:** 10.1039/c9ra00285e

**Published:** 2019-03-26

**Authors:** Le Lam Son, Nguyen Duc Cuong, Tran Thi Van Thi, Le Trung Hieu, Do Dang Trung, Nguyen Van Hieu

**Affiliations:** University of Sciences, Hue University 77 Nguyen Hue Hue City Vietnam; School of Hospitality and Tourism, Hue University 22 Lam Hoang Hue City Vietnam nguyenduccuong@hueuni.edu.vn; Department of Basics Science, University of Fire Fighting and Prevention 243 Khuat Duy Tien, Thanh Xuan Hanoi Vietnam Trungdo81@gmail.com; Faculty of Electrical and Electronic Engineering, Phenikaa Institute for Advanced Study (TIAS), Phenikaa University Yen Nghia, Ha-Dong District Hanoi 10000 Vietnam hieu.nguyenvan@phenikaa-uni.edu.vn; Phenikaa Research and Technology Institute (PRATI), A&A Green Phoenix Group 167 Hoang Ngan Hanoi 10000 Vietnam

## Abstract

Biopolymer template synthesis has attracted extensive interest for fabricating highly porous metal oxide nanostructures. In this report, a green template-based approach for the synthesis of three-dimensional (3D) NiO nanostructures assembled from porous NiO nanoplates is introduced using a konjac glucomannan (KGM) template. The Ni–KGM composites, which were formed by the immersion of KGM nanofibrils in nickel nitrate solution, were annealed in air at 600 °C to obtain the highly porous NiO nanoplates. The KGM nanofibrils were used as a sacrificial template, which was combusted at a high temperature for the formation of the porous nanostructures. The gas sensor properties of the porous NiO architecture were systematically investigated with four reduced gases including hydrogen sulfide, ammonia, carbon monoxide and hydrogen. The results indicate that the porous NiO nanoplates show a good detection of hydrogen sulfide with a rapid response and recovery speed at low concentrations.

## Abstract 2

Biopolymer template synthesis has attracted extensive interest for fabricating highly porous metal oxide nanostructures.

## Graphical abstract

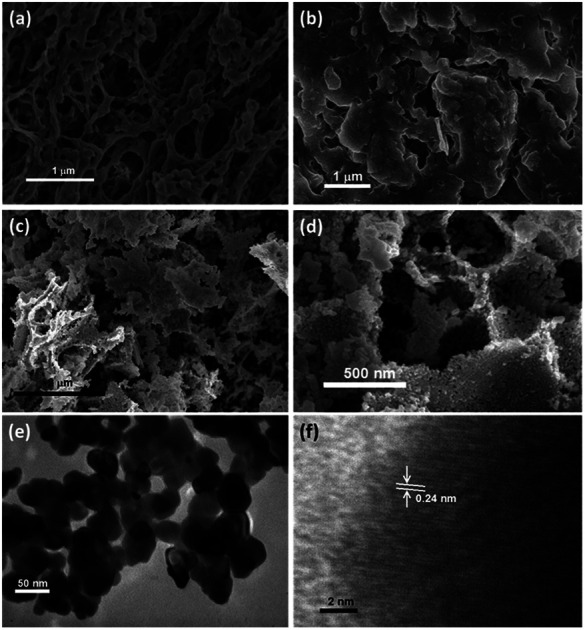

## 1. Introduction

Highly porous materials are a subject of great interest due to their unique architecture including the integration of a high specific surface area, hierarchical porous organization, and interfacial junctions.^1^ Among various strategies for the design of novel nano-architectures to date, using natural polymers as sacrificial templates plays an important role for creating highly porous and lightweight materials because this approach is inexpensive, environmentally friendly and facile for application for many materials.^2^ Natural polymers usually possess a lot of functional groups that can interact with metal ions through electrostatic attractions to form coordination complexes^3,4^ in which the natural hosts can function as a template and transfer their hierarchical scaffolds to generate porous materials. Kim *et al.*^5^ have successfully synthesized porous network TiO_2_ nanoribbons that maintained the natural geometries of peptide precursors. Walsh *et al.*^2,6^ introduced a novel method to fabricate a series of porous metallic, metal oxide and composite sponges using the natural biopolymers dextran and xyloglucan as framework-generating agents. Song *et al.*^7^ reported the successful fabrication of mesoporous TiO_2_ with high exposure of the (001) faces using a cellulose nanocrystal template. Nguyen *et al.*^8^ successfully converted hierarchical chitosan nanofibril templates into lightweight γ-Al_2_O_3_ aerogels.

Konjac glucomannan (KGM), which is a renewable natural polymer, is extracted from *Amorphophallus konjac* tubers.^9^ The KGM polymer chain, which is constructed of β-1,4 linked d-mannose and d-glucose, possesses a lot of hydroxyl groups and a small amount of acetyl groups attaching randomly to the C-6 position.^10^ Taking the intrinsic advantage of the unique 3D skeleton structure of KGM, some highly porous and lightweight materials based on KGM have been fabricated such as 3D network structures of KGM/graphene oxide sponges,^11^ foam-like KMG/montmorillonite,^12^ Fe and Mn oxides/KGM aerogels,^13^ and artificial nacre-like KGM–montmorillonite–Ag composite films.^14^ Thus, designing novel highly porous architectures based on KGM biopolymer templates is a potential strategy to find new applications.

Nickel oxide (NiO), which is one of the important metal oxides, has attracted significant attention because of its low cost, good safety, environmental benignity, and unique chemical and physical properties.^15^ The highly porous NiO nanostructures usually exhibit new characteristics or improve existing performances.^16^ For example, the NiO nanorings showed an unexpected catalytic feature for carbon monoxide oxidation.^17^ The hierarchical porous NiO nanotubes exhibited remarkable supercapacitor performance with high capacitance and good cycle life.^18^ The NiO nanotubes showed great antibacterial activities in comparison with the NiO nanoflowers and commercial NiO.^19^ Some natural templates have been used to design NiO-based porous nanostructures such as nanocrystalline cellulose,^20^ eggshell membrane,^21^ lotus pollen grains,^22^ guar gum,^23^ starch,^24^ and pinewood.^25^ However, to the best of our knowledge, there have seldom been reports of using natural KGM as a sacrificial support for designing highly porous NiO nanostructures in today's literature. Here, we report a novel synthetic route to 3D NiO nanostructures using biotemplating KGM nanofibrils and their gas sensing characteristics were systematically investigated.

## 2. Experimental

### 2.1. Synthesis of 3D NiO nanostructures

Konjac glucomannan (KGM, Shimizu Chemical Co., Japan), nickel nitrate (Ni(NO_3_)_2_, Sigma-Aldrich) and C_2_H_5_OH (96 vol%, Quangzi, China) were purchased and used without further purification.

**Scheme 1:**
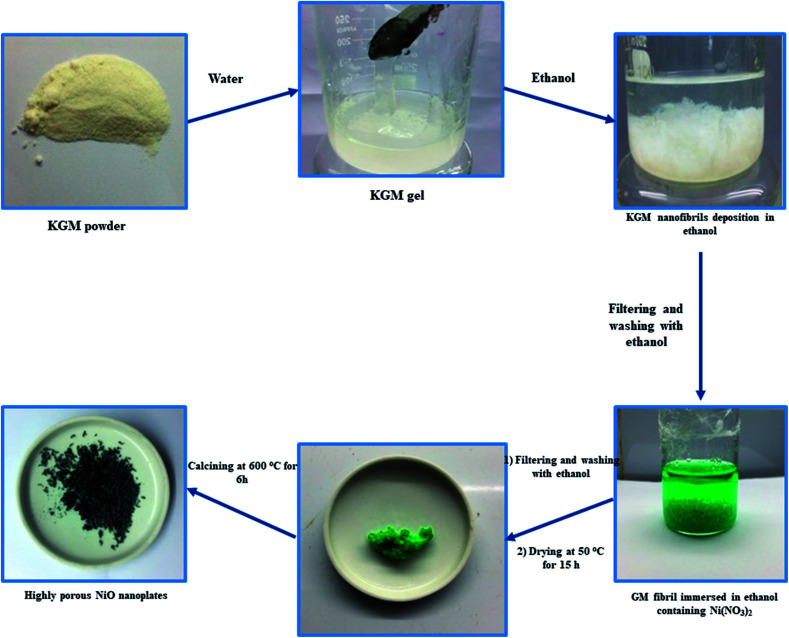
Schematic diagram of the producing process of the porous NiO nanoplates.

In a typical experiment, 1 g of KGM was dissolved in 50 mL of deionized water by magnetic stirring for 30 min to form a KGM gel. After, the obtained KGM gel was mixed with 50 mL of ethanol for 10 min to form KGM nanofibril deposition. The KGM nanofibrils were taken out and washed with ethanol several times. After that, KGM nanofibrils were immersed in 30 mL of ethanol solution containing 0.03 mol Ni(NO_3_)_2_ for 24 h. The Ni^2+^ absorbed KGM nanofibrils were taken out and washed with ethanol several times. The Ni–KGM composites were dried at 50 °C for 15 h in an oven and then calcined at 600 °C for 6 h with a heating rate of 2 °C min^−1^ to obtain highly porous NiO nanoplates. The schematic diagram of the production of porous NiO nanoplates is shown in Scheme 1.

### 2.2. Material characterization

X-ray diffraction patterns of the products were performed using a Bruker D8 Advance X-ray diffractometer. Scanning electron microscopy (SEM) and elemental analysis and energy dispersive X-ray spectroscopy (EDX) were recorded by a JSM-5300LV instrument. Transmission electron microscopy (TEM) and high-resolution TEM (HRTEM) were measured using JEOL JEM 1230. Thermogravimetric analysis (TG) and differential thermal analysis (DTA) were conducted using a Labsys TG/DTA-SETARAM simultaneous thermogravimetric analyzer with a heating rate of 2 °C min^−1^ in air environment. The Nicolet 6700 FTIR Spectrometer was used to measure the infrared spectra of the samples. Nitrogen adsorption/desorption isotherms was used to analyze the specific surface area and pore size distribution of the highly porous NiO nanoplates. The specific surface area (*S*_BET_) and pore size distribution of the nanomaterials were calculated according to the Brunauer–Emmett–Teller (BET) equation and the Barrett–Joyner–Halenda (BJH) method, respectively.

### 2.3. Gas sensing characterizations

Thick film technique was used to test the gas sensing features of the porous NiO nanoplates. Briefly, Ni^2+^ absorbed KGM nanofibrils were dispersed in ethanol to form a slurry. The obtained suspension was drop-cast onto an interdigitated electrode substrate using a 1 mm diameter needle. The prepared sensors were annealed at 600 °C for 6 h to complete the transformation of Ni–KGM nanofibrils to highly porous NiO nanoplates as well as increasing the adhesion of the sensing materials into the electrode. The highly porous NiO nanoplates were tested to detect some reduced gases including H_2_S (0.5–20 ppm), NH_3_ (12.5–250 ppm), H_2_ (25–250 ppm), and CO (5–200 ppm) at various operating temperatures. A homemade set-up employed a flow-through with a standard flow rate of 200 sccm (standard cubic centimeters) for the reference and target gases. Balance gases (0.1% in air) were purchased from the Air Liquid Group (Singapore). The resistance of the sensors during the sensing measurement was automatically recorded using Keithley 2602, controlled by a computer through a software program.^26,27^ The details of the sensing measurement protocol are presented in Fig. S1.† The sensitivity of the nanosensor was defined as *S* = *R*_gas_/*R*_air_, where *R*_gas_ and *R*_air_ were assigned to the resistance of the sensor in ambient target gas and in air, respectively. Response time (*τ*_res._) and recovery time (*τ*_recor_) were determined as the time for the resistor to change from *R*_air_ to *R*_air_ + 90%(*R*_gas_ − *R*_air_) and *R*_gas_ to *R*_gas_ − 90%(*R*_gas_ − *R*_air_), respectively.

## 3. Results and discussion

**Fig. 1:**
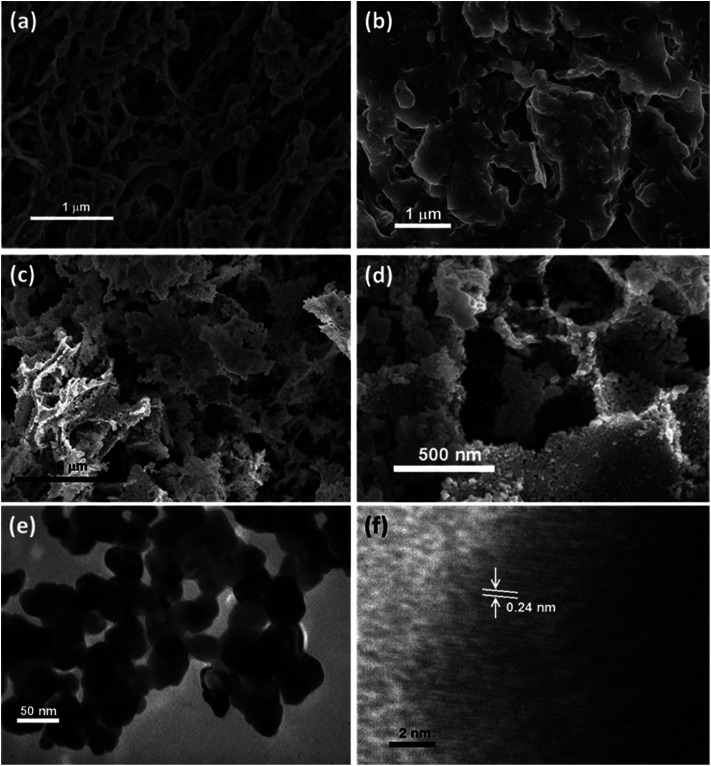
(a) SEM image of the KGM; (b) SEM image of the Ni–KGM composites; (c and d) SEM images, and (e) TEM image and HRTEM image of the porous NiO nanoplates (f).

Scheme 1 indicates that the KGM was easily dissolved in water to form a homogeneous colloidal suspension that is deposited and swelled in ethanol solvent. The SEM images (Fig. 1(a) and S2(a)†) reveal that the KGM template exhibits a highly porous open network through the interconnected fibrils. To form the Ni–KGM composites, the KGM nanofibril template was immersed in nickel nitrate solution. Visibly, the milk-white color of KGM transferred to a green color after the immersing process, indicating the successful absorption of Ni^2+^ ions on the KGM template (Scheme 1 and Fig. S3†). The SEM image in Fig. 1(b) shows that the Ni–KGM nanofibers were aggregated together to form the plates. The as-prepared Ni–KGM nanofibrils were used as the precursor to generate hierarchical NiO nanostructures by annealing in air. As can be seen in Fig. 1(c and d), the calcined sample shows highly porous nanostructures. The 3D nanostructures with many big holes are formed by self-assembly of the porous NiO nanoplates, in which the individual NiO nanoplates with a thickness of ∼50 nm (Fig. S2(b)†) are formed through NiO nanoparticle agglomeration. Many bright pores were observed in the TEM image (Fig. 1(e)), which further demonstrates the highly porous nature of the NiO nanoplates. The HRTEM image in Fig. 1(f) shows the clear lattice spacing between the adjacent planes of 0.24 nm, relating to the (111) planes of the NiO crystal.

**Fig. 2:**
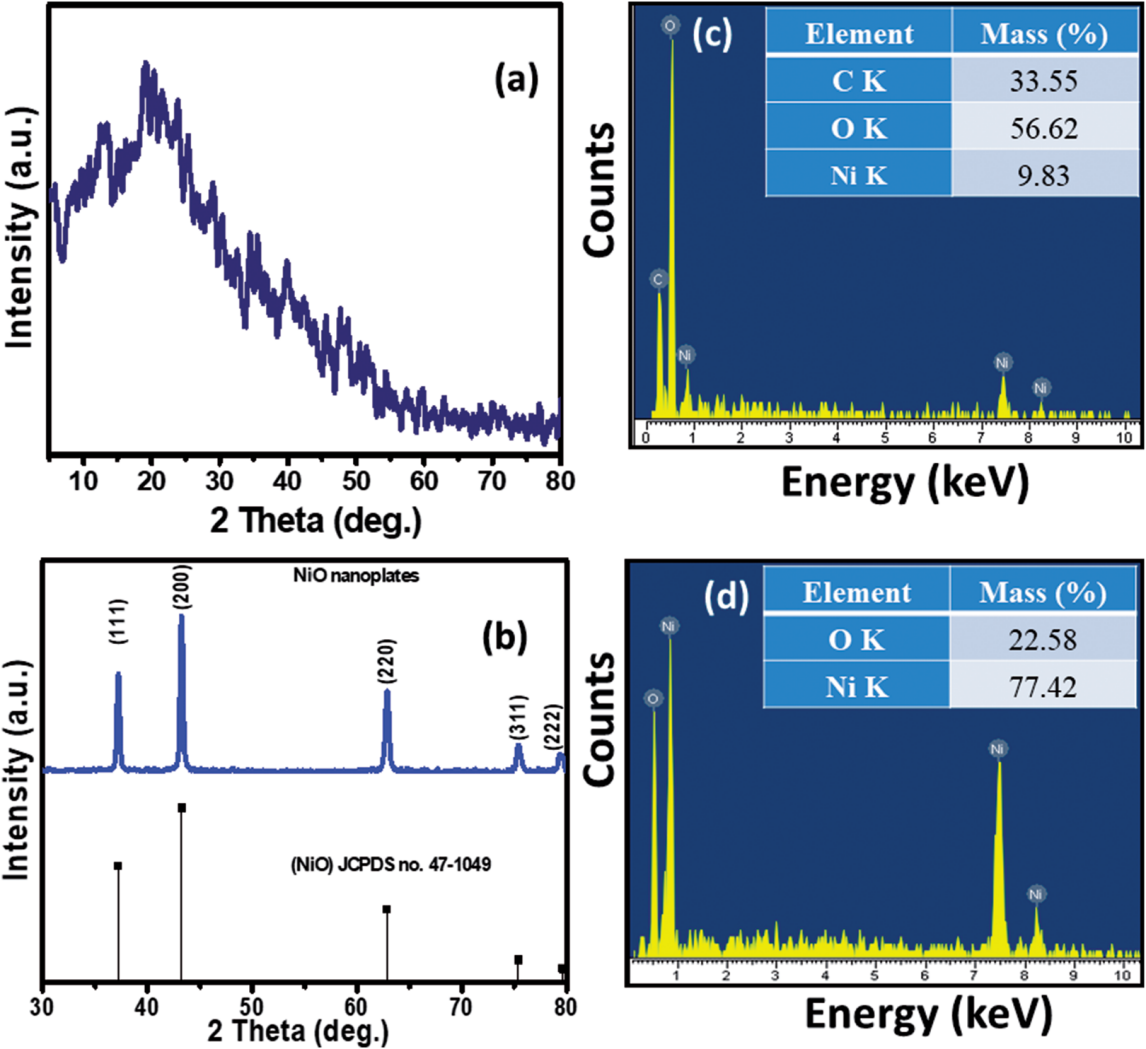
XRD patterns of the Ni–KGM composites (a) and the porous NiO nanoplates (b); EDX spectra and respective mass percentages of the elements of the Ni–KGM composites (c) and the porous NiO nanoplates (d).

The XRD patterns of the Ni–KGM nanofibrils and calcined porous NiO nanoplates are exhibited in Fig. 2(a) and (b), respectively. The XRD patterns of Ni–KGM (Fig. 2(a)) and KGM (Fig. S4†) are the same with a broad dispersion peak at 2*θ* ∼ 19° and no obvious diffraction peaks. The results indicate that both KGM and Ni–KGM have a low degree of crystallinity. The XRD spectra of the calcined NiO nanomaterials (Fig. 2(b)) show five diffraction peaks at 37.22°, 43.28°, 62.84°, 75.38°, and 79.37° corresponding to the (111), (200), (220), (311) and (222) planes, respectively. These diffraction peaks can be perfectly related to the face centered cubic phase of NiO (JCPDS no. 47-1049). High intensity and sharp diffraction peaks in the XRD spectra indicate that the porous NiO nanoplates have good crystallinity. Furthermore, no other peaks relating to impurities such as nickel carbide or pure nickel were observed, indicating the complete thermal conversion of the Ni–KGM precursor into porous NiO nanoplates.

The chemical compositions of Ni–KGM and the porous NiO nanomaterials were analyzed using EDX (Fig. 2(c and d)). The strong peaks of three elements including Ni, C and O were observed in the EDX spectra of the Ni–KGM nanofibrils (Fig. 2(c)), confirming that the Ni ions have been successfully incorporated into the KGM fibers. The as fabricated Ni–KGM composites contain ∼9.83 wt% nickel (inset of Fig. 2(c)). The EDX analysis of the calcined sample (Fig. 2(d)) shows the presence of nickel and oxygen only, with a calculated Ni/O atomic ratio of ∼1.0. Both the XRD and EDX results indicate that the calcined sample is pure NiO.

**Fig. 3:**
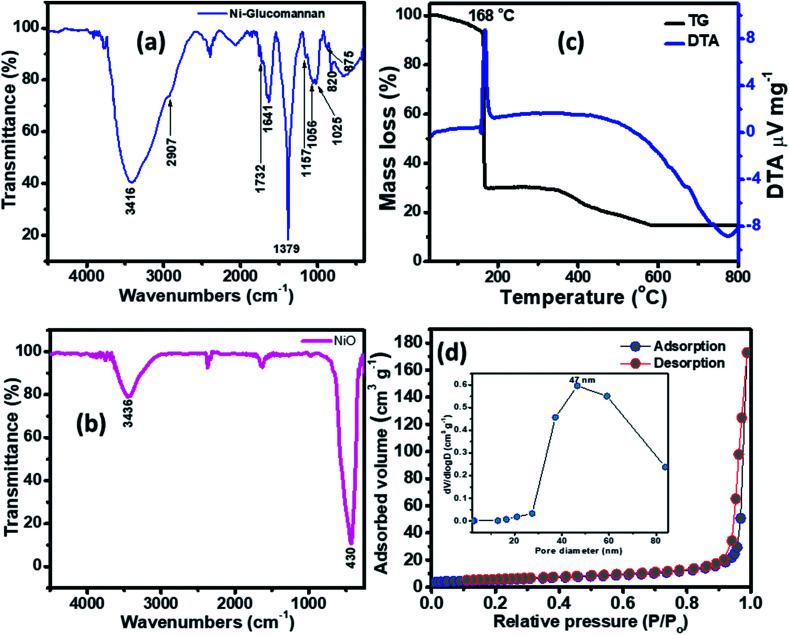
FTIR spectra of the Ni–KGM composites (a) and the porous NiO nanoplates (b); TG-DTA curves of the Ni–KGM composites (c); and the nitrogen adsorption/desorption isotherm of the porous NiO nanoplates (d).

The FTIR of the Ni–KGM nanofibrils and the calcined NiO materials are shown in Fig. 3(a) and (b). For the FTIR spectra of Ni–KGM (Fig. 3(a)), almost all the characteristic peaks of KGM are clearly observed. The wide band around 3416 cm^−1^ is related to the hydroxyl stretching vibrations of polysaccharide.^28^ The band at 2907 cm^−1^ corresponds to the –CH_2_– stretching vibration while the peaks at 1379 cm^−1^ and 1056 cm^−1^ are confirmed to be two C–H bending modes.^29^ The small peak at 1732 cm^−1^ is revealed to be a C=O stretching vibration, whereas the peak at 1641 cm^−1^ is due to adsorbed water. The C–O–C bending stretching vibrations in the ether groups and in the pyranose rings exhibit at 1157 cm^−1^ and 1025 cm^−1^, respectively.^29^ Peaks from 875 and 825 cm^−1^ are typical for β-glucosidic and β-mannosidic linkages, respectively.^30^ The FTIR of the calcinated sample (Fig. 3(b)) shows that all the characteristic bands of KGM are not observed, in which the strong band at 430 cm^−1^ is assigned to the Ni–O bending stretching vibration. The result indicates that the Ni–KGM composites are successfully transferred to the porous NiO nanoplates after the annealing process.

The thermal decomposition behavior of the Ni–KGM nanofibrils was analyzed by TGA-DTA, and the result is presented in Fig. 3(c). As can be seen in the TG curve, three main weight loss steps are observed. The first weight loss of ∼7 wt% at 30–150 °C may correspond to the liberation of adsorbed solvent.^31^ The second step with a total weight loss of ∼64 wt% at 150–300 °C may be attributed to the decomposition and combustion of KGM associated with an exothermic peak at 168 °C in the TGA curve.^32^ The final weight loss of ∼15 wt% in the temperature range 300–600 °C may be related to the complete oxidation of KGM as well as the formation of the NiO phase. No weight loss is determined beyond 600 °C, demonstrating that the Ni–KGM nanofibrils transfer successfully into the final monophasic NiO.

The textural characteristics of the as synthesized porous NiO nanoplates were analyzed by the nitrogen adsorption–desorption isotherm measurements (Fig. 3(d)). The N_2_ adsorption–desorption isotherm of the 3D NiO nanostructures (Fig. 3(d)) exhibits a type IV with H1 hysteresis loops that are typical for the macro–mesoporous structure.^33^ The BET surface area of the sample is 18 m^2^ g^−1^. The BJH pore size distribution (inset of Fig. 3(d)) of sample shows both a mesoporous and macroporous region with average diameter at 47 nm. The meso- and macro-scale pores may be formed by the agglomeration of NiO nanoparticles^34^ and assembly of the nanoplates.^8^ The novel porous NiO nanostructures usually give rise to the unique gas sensing properties originating from the defective sites on their surface.

**Fig. 4:**
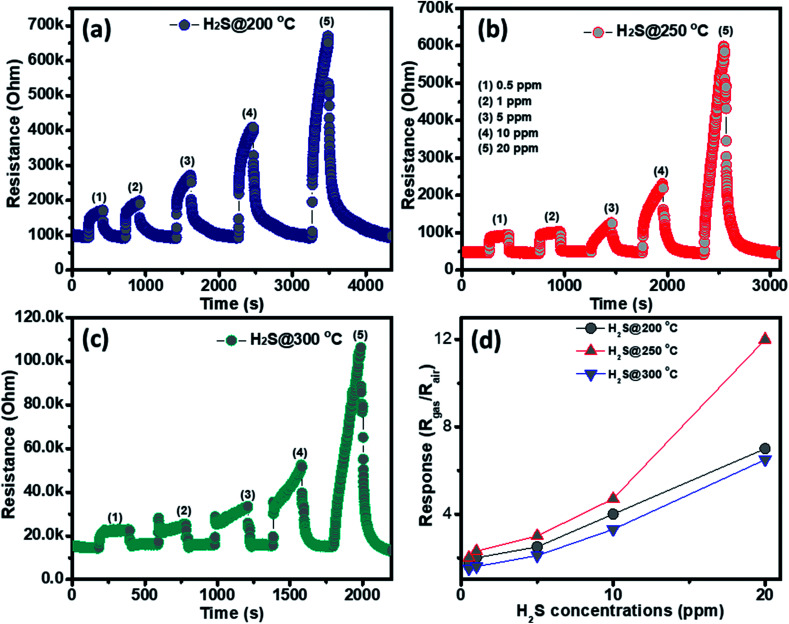
Transient resistance of the porous NiO nanoplates to H_2_S gas at 200 (a), 250 (b) and 300 °C (c) and response as a function of gas concentration (d).

The gas sensing behavior of the as fabricated mesoporous NiO nanoplates was tested with H_2_S, NH_3_, CO and H_2_ gases. The sensors were measured with H_2_S and NH_3_ gases at various operating temperatures to find the optimum conditions because the gas response is highly dependent on the working temperature. This relationship can be explained from the competition between the surface reaction and Knudsen diffusion^35^ that has already been demonstrated in some of our previous reports.^36,37^Fig. 4(a–c) shows the resistance curves of the sensor to various concentration of H_2_S under different temperatures. It can be clearly seen that the porous NiO nanoplates-based sensor can detect H_2_S at a very low concentration for all measuring temperatures. The resistance of the sensors increases after exposure to a variety of H_2_S gas concentrations (0.5–20 ppm), and recovery to the near initial stage when exposed to dry air, which shows good reversibility. The sensor response as a function of measuring temperature to various H_2_S concentrations is exhibited in Fig. 4(d). The response increases as the working temperature rises from 200 to 250 °C and reaches a maximum at 250 °C, and then decreases at higher operating temperatures (300 °C). At the optimum temperature, the responses of the porous NiO nanoplate sensor increase quickly from 2 to 12 with increasing H_2_S concentration ranging from 0.5 to 20 ppm. The good linear dependence indicates that the porous NiO nanoplates are a good promising material for H_2_S sensors. The NiO nanostructures-based sensors have been widely investigated for excellent detection of volatile organic compounds^38–44^ but there are limited reports about their hydrogen sulfide sensing characteristics.^45–47^

**Fig. 5:**
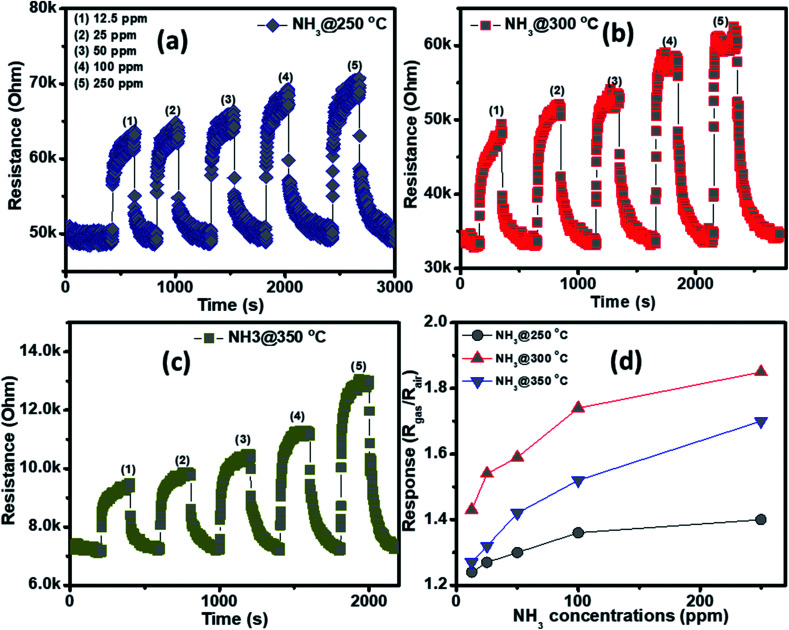
Transient resistance of the porous NiO nanoplates to NH_3_ gas at 250 (a), 300 (b) and 350 °C (c) and response as a function of gas concentration (d).

The NH_3_ sensing characteristics of the porous NiO nanoplates are shown in Fig. 5. In the concurring H_2_S results, the initial resistance of the sensor increases when the NH_3_ gas is injected into the gas chamber. After five cycles between the ammonium gas and dry air, the base resistance of the porous NiO sensor can approximately recover its initial value at all working temperatures (Fig. 5(a–c)). This result indicates that the porous NiO nanoplate sensor shows good reversibility. However, the resistance of the nanosensor increases slowly with the rise of ammonium concentrations (12.5–250 ppm) and its best working temperature is 300 °C (Fig. 5(d)). At the optimum temperature, the responses of the sensor only increase slightly from 1.43 to 1.85 with the increased NH_3_ concentrations, indicating that the porous NiO nanoplates have a low sensitivity to NH_3_ gas.

**Fig. 6:**
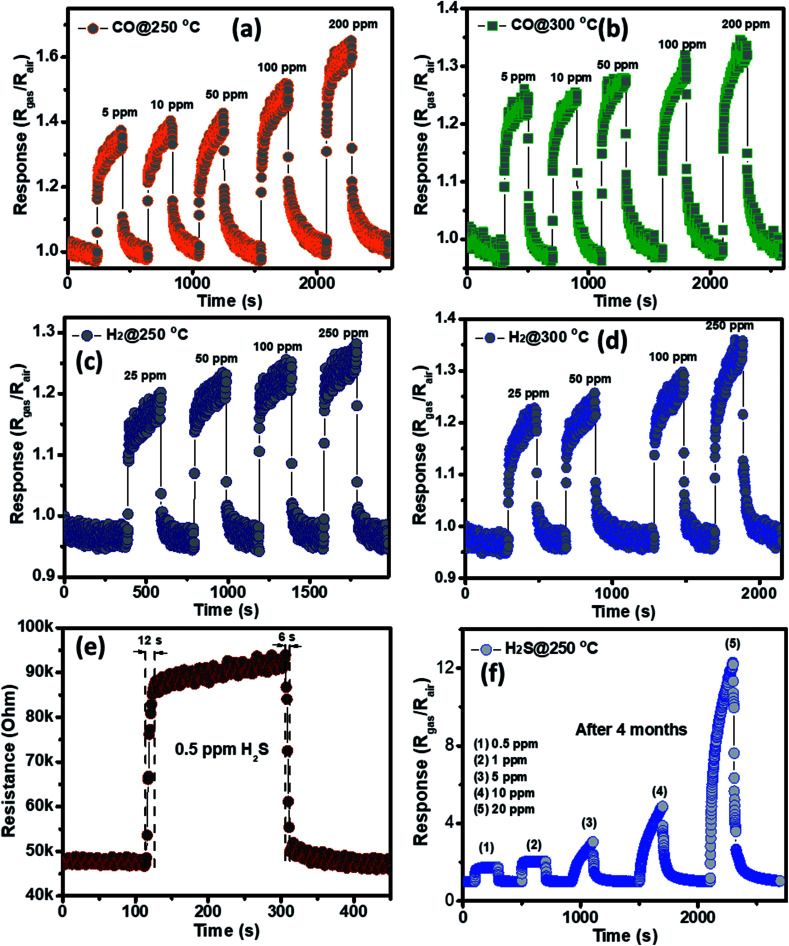
Transient response of porous NiO nanoplates to CO (a and b) and NH_3_ (c and d) gases at different working temperatures; response–recovery times of the NiO nanoplates sensors to 0.5 ppm of H_2_S at optimum temperature (e); and the response to different gas H_2_S concentrations at 250 °C measured after 4 months (f).

The selectivity of the porous NiO nanoplates sensors were also tested to other reduced gases, including carbon monoxide (5–200 ppm) and hydrogen (25–250 ppm) (Fig. 6(a–d)) at 250 and 300 °C. As can be seen in Fig. 6(a–d), the sensor response increases slightly with the increase of gas concentration at both working temperatures. At the highest concentration for both gases, their response values are not more than 1.6. The results show that the porous NiO nanoplates have a good selectivity to H_2_S. Fig. 6(e) and S5(a)† show the response and recovery times of the sensor toward low H_2_S concentrations (0.5 and 1 ppm) at 250 °C, respectively. The porous NiO nanoplates exhibit a short response and recovery times at low hydrogen sulfide concentrations. However, their response times at higher concentrations (5–20 ppm) are relatively long (Fig. S5(b)†) when the sensor resistance reaches to near the saturation state with the testing time of about 200 s. The recovery time also increases significantly with the rising of H_2_S concentration. At 20 ppm H_2_S, the recovery time increases about 15-fold in comparison with that of 0.5 ppm (Fig. 6(e) and S5(b)†). For practical applications at high H_2_S concentrations, this problem should be improved in our future investigations. To verify the stability of the sensors based on porous NiO nanoplates, the sensor, which was placed in an ambient room for 4 months, was re-used to test with various H_2_S concentrations at 250 °C. As shown in Fig. 6(f), the sensor presented similar response values in comparison with that of the fresh sensors even after 4 months. These results demonstrate that the sensor has a good stability and it can satisfy the H_2_S gas detection in practice.

For a typical p-type semiconducting metal oxide, the majority of the carriers of the porous NiO nanoplates are holes. The sensing mechanism relates dramatically to the increase and decrease of the hole concentration on the sensing surface, which result from chemisorbed oxygen and oxidation–reduction reactions as well as the effective diffusion of gas.^48^ When the porous NiO nanosensor is exposed to dry air, O_2_ molecules trap their conduction band electrons to form oxygen ions (O^−^, O_2_^−^, and O^2−^).^49^ This process leads to the highly conductive state of the sensors because of the richness of holes. Once reduced gases were injected, the target gas molecules were oxidized by the oxygen species. These reactions release electrons that rapidly neutralize the holes, which causes the increasing sensor resistance.^50^ Thus, the sensitivity and selectivity of the NiO sensors may not only depend on their intrinsic properties but also may relate to the morphologies. Li^51^ found that NiO nanowires showed good detection toward HCHO because of their one dimensional structure high aspect ratio and large specific surface area. Lv *et al.*^52^ demonstrated that the enhanced ethanol gas sensing of octahedral NiO particles was attributed to the exposed {111} facets of the octahedron. H_2_S molecules can be adsorbed on the NiO surface through interaction with oxygen ions as well as direct interaction by chemical affinity between Ni and S.^53,54^ Furthermore, the nanostructures with large pore sizes have higher diffusion constants.^55^ Thus, we believe that the high sensitivity and good selectivity toward H_2_S of the porous NiO nanoplate may relate to two reasons: (i) the enhanced adsorption of hydrogen sulfide molecules on the NiO surface by direct interaction and (ii) the large pore size system of the NiO nanostructures can allow most gas H_2_S molecules to diffuse easily inside the deeper region of the porous architectures and then lead to an increasing sensor response.

## 4. Conclusions

In summary, porous NiO nanoplates have been successfully fabricated through a green route using a natural KGM polymer template. KGM nanofibrils were used as a sacrificial template, which was combusted at a high temperature to form porous nanostructures. The hierarchical porous NiO architectures were formed by the aggregation of primary nanoplates, in which individual NiO nanoplates with a thickness of ∼50 nm are formed through NiO nanoparticle agglomeration. The porous NiO nanoplate-based sensor shows good detection of H_2_S at low concentrations with fast response and recovery times.

## Conflicts of interest

There are no conflicts to declare.

## Supplementary Material

RA-009-C9RA00285E-s001
