# Supplementary material for: Konjac glucomannan-templated synthesis of three-dimensional NiO nanostructures assembled from porous NiO nanoplates for gas sensors
Source: RSC Adv. 2019 Mar 26;9(17):9584–93. doi: 10.1039/c9ra00285e (PMC9062158; doi:10.1039/c9ra00285e)

Electronic Supplementary Material (ESI) for RSC Advances.
This journal is © The Royal Society of Chemistry 2019

Electronic Supplementary information (ESI)

Fig. S1. Design of a gas sensor by drop-cast of an ethanol dispersion of Ni-KGM composites on aninterdigitated Pt electrode substrate followed by heating under air to form a porous NiO nanoplate layer-deposited electrode, (b) Photo of gas testing chamber for gas sensing measurement, and (c) Diagram of the working system of gas sensor.

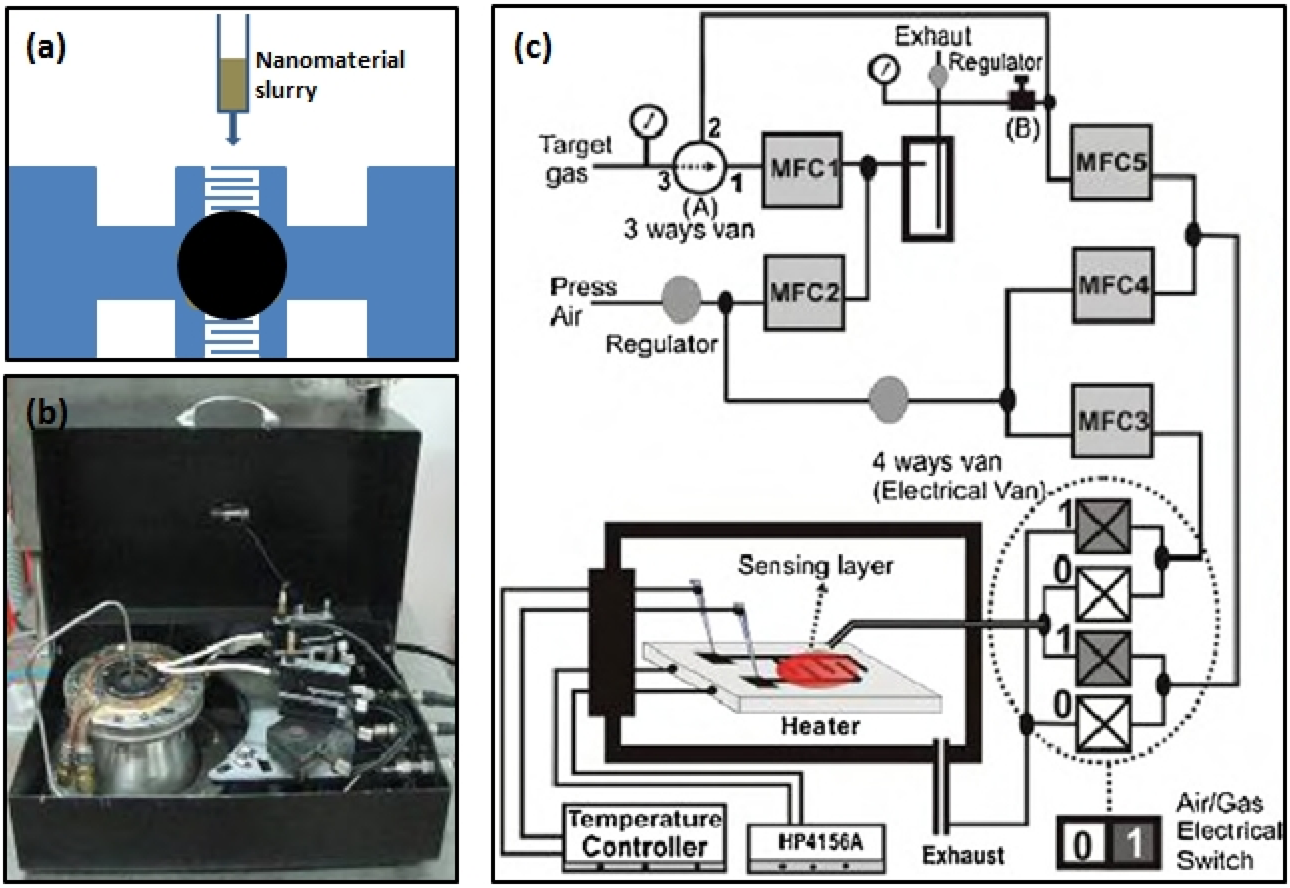

Fig. S2. Low magnification SEM image of konjac glucomannan (a) and high magnification SEM image of porous NiO nanoplates.

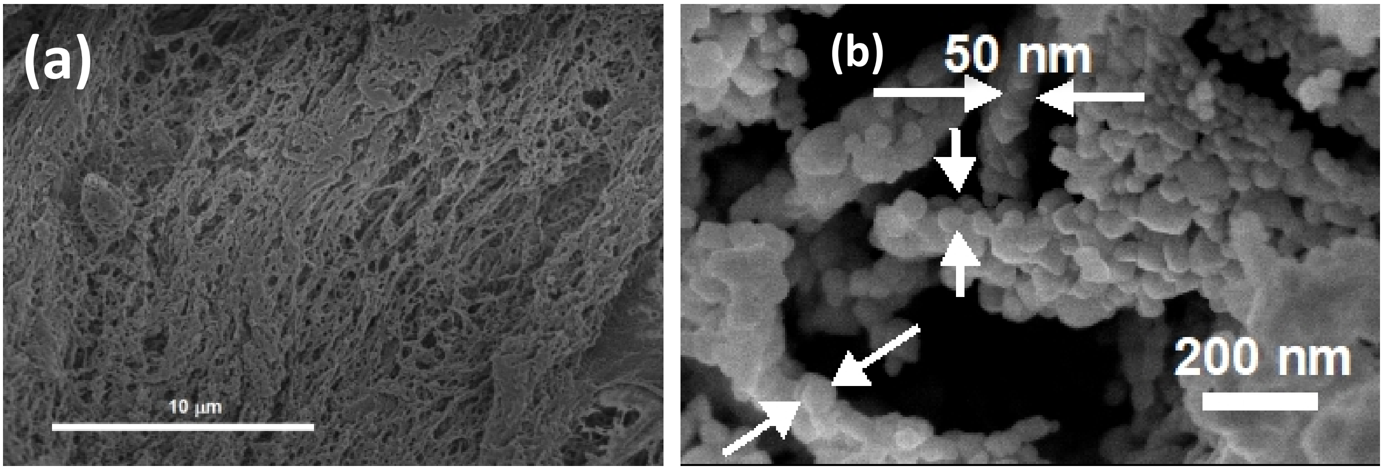

Fig. S3. Photograph of Ni-KGM composites.

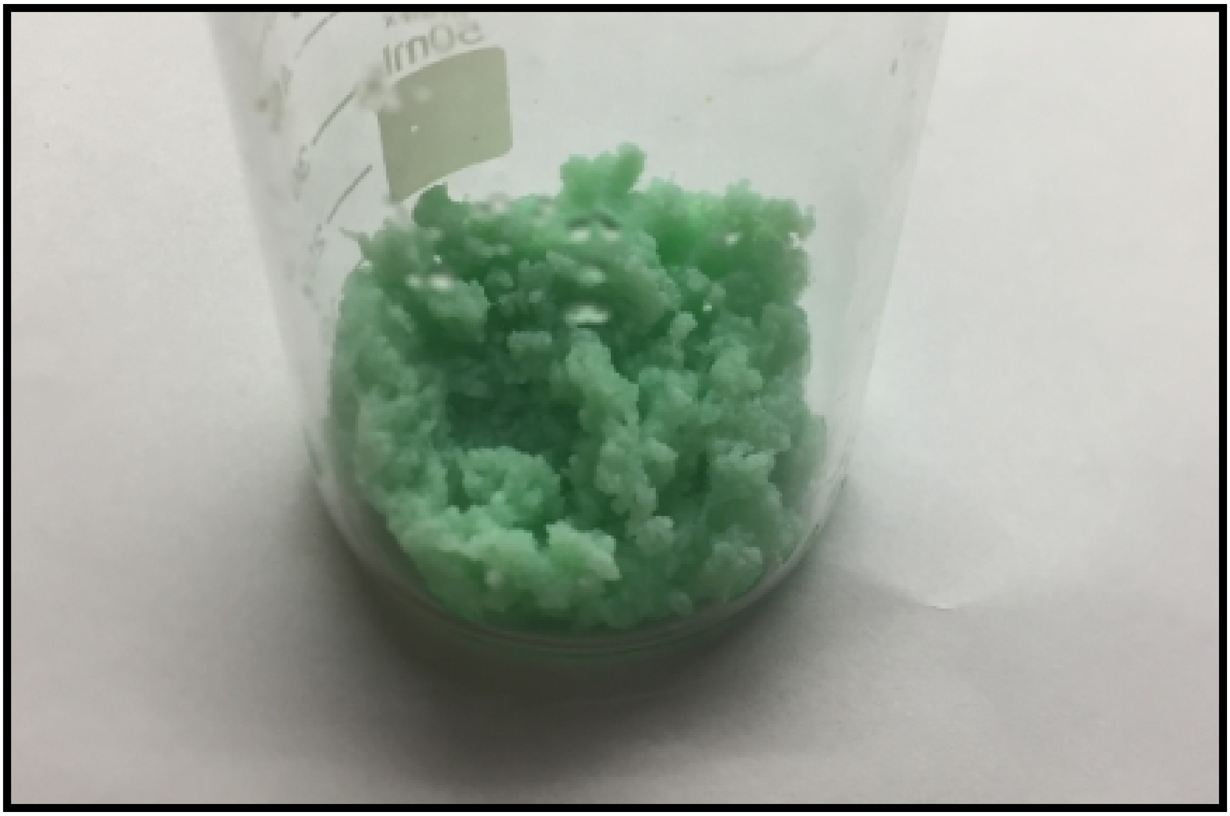

Fig. S4. XRD pattern of KGM.

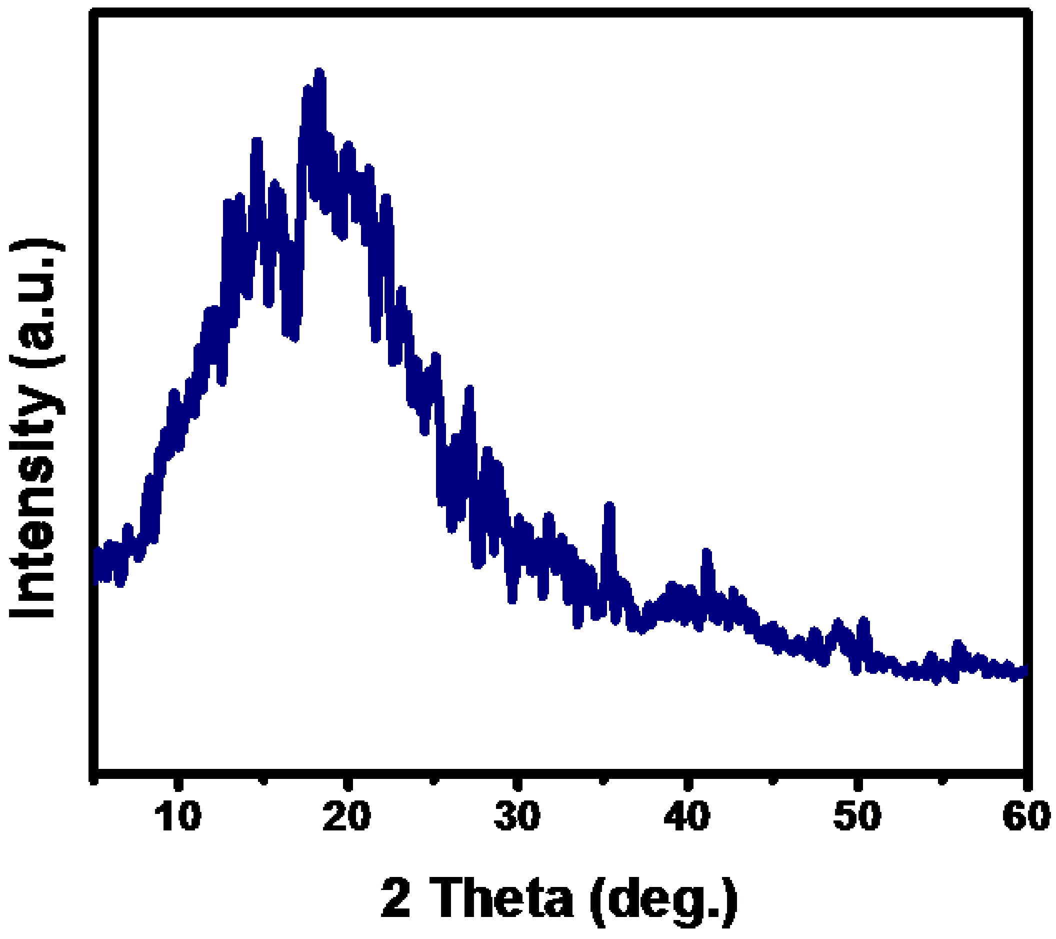

Fig. S5. Response and recovery times of the NiO nanoplate sensors toward 1 ppm of H_2S (b) and recovery times of the NiO nanoplate sensors toward higher concentrations of H_2S (b) at optimum temperature.

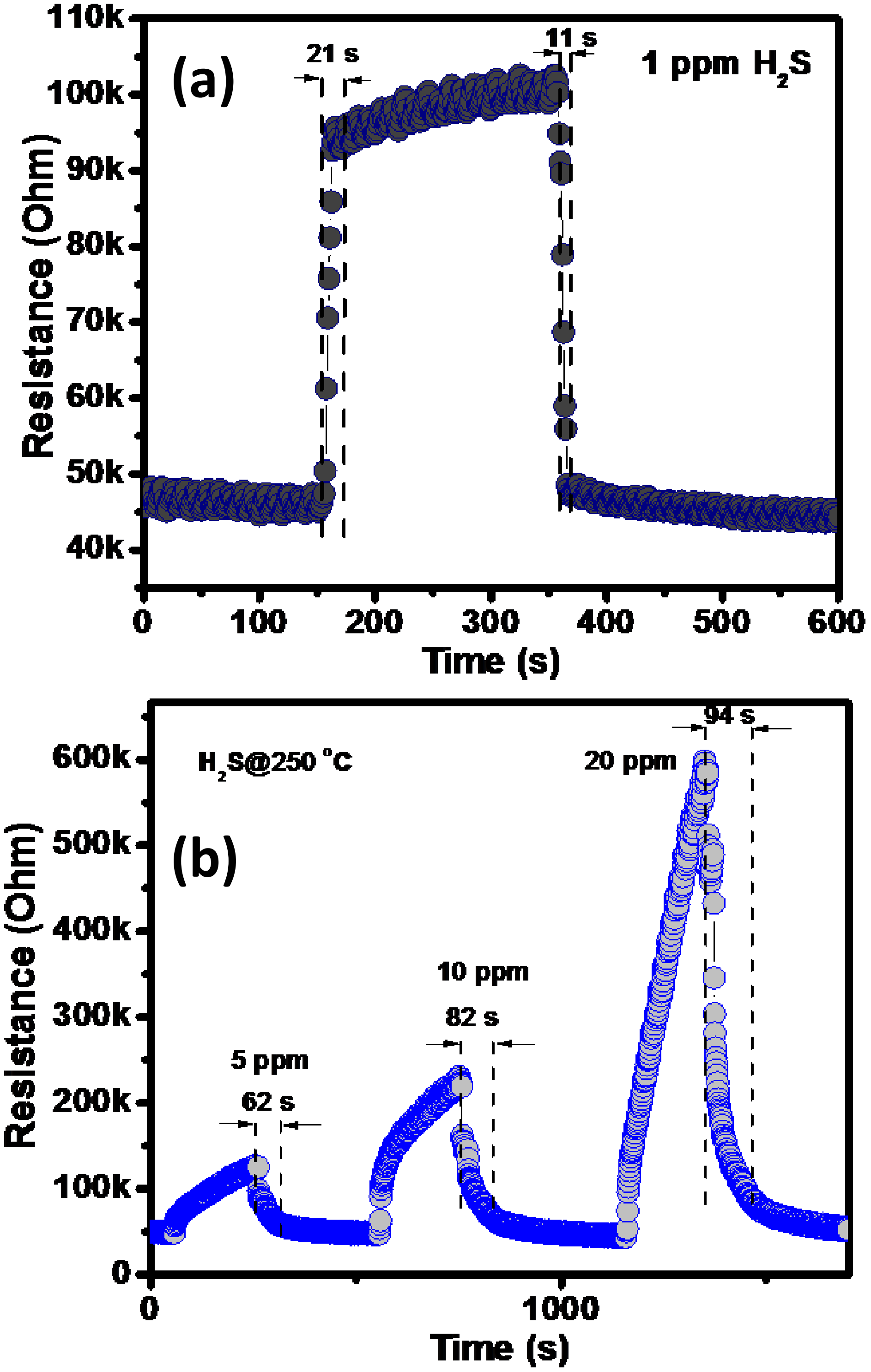

Supplement: RA-009-C9RA00285E-s001 [file RA-009-C9RA00285E-s001.pdf]
